# “Sanitary measures, social distancing, safety”: The evolution of Swiss hoteliers’ Covid-19 communication through three snapshots

**DOI:** 10.1177/14673584231162279

**Published:** 2023-03-13

**Authors:** Laura Zizka, Meng-Mei Chen

**Affiliations:** EHL Hospitality Business School//HES-SO University of Applied Sciences and Arts Western Switzerland, Switzerland; EHL Hospitality Business School//HES-SO University of Applied Sciences and Arts Western Switzerland, Switzerland

**Keywords:** Covid-19 pandemic, websites, situational crisis communication theory, crisis communication evolution, visualization

## Abstract

Since the first reports of the Covid-19 virus in December 2019, the tourism industry has struggled to find solutions to this unprecedented crisis. During crises, organizational learning can develop crisis management and communication skills while enhancing organizational resilience in coping with crises. This research examines whether organizational learning for communicating during crises occurred in the Swiss hotel industry in the past 2 years. By tracking and visualizing the messages communicated by Swiss hotels on their websites, this study analyzes the communication strategies employed by hoteliers over the past 20 months through thematic analysis, co-occurrence analysis, and Situational Crisis Communication (SCCT) strategies. The results of this study identified the evolution of communication strategies over time. Specifically, ingratiation, corrective action, transferring, victimization, and justification are the five key strategies. This study also visualizes the crisis responses in concept maps in three snapshots (June 2020, June 2021, and February 2022). The concepts and colors of the visualization provide a different perspective on the evolution of crisis communication over the past 2 years. This study contributes to academia and practitioners by demonstrating the evolution of crisis communication messages through both the granular analysis of SCCT strategies and the bird’s-eye view of themes and concepts.

## Introduction

Organizational learning is a critical crisis management function (Liu-Lastres et al., 2020). During crisis times, organizational learning can enhance crisis management and communication skills while enhancing organizational resilience in coping with crises (Liu-Lastres et al., 2020). The Covid-19 pandemic has had an adverse effect on tourism. According to the World Tourism Organization (2022), the world tourism industry barely improved in 2021 compared to 2020, with all indicators resting below pre-pandemic levels. Further, industry professionals have predicted that they do not expect a full recovery before 2024 (Pervilhac and Draser, 2021). Hence, it is critical to study if the tourism industry has strengthened its resilience in the past 2 years.

Like many destinations, Switzerland relies on international travelers who greatly contribute to the country’s gross domestic product (GDP). According to the Federal Statistic Office, the Swiss hotel sector registered a 40% drop in overnight stays in 2020 compared with 2019. This disappearance of foreign travelers led the Switzerland Tourism body to name 2020 its “annus horribilis” (Federal Statistical Office, 2021). However, according to data published by Central Bank, Swiss tourism revenues rebounded in 2021 with an increase of 3217 million CHF (3480 million USD) in the third quarter of 2021 (Central Bank, 2021). One explanation was the explosion of domestic demand in 2021, as 21 million domestic nights were recorded, 27.9% more than in 2020 and 17% more than in pre-pandemic levels (Doyle, 2022).

According to the Federal Statistical Office (FSO), 29.6 million overnight stays, both domestic and international, were recorded by Swiss hotels in 2021 – 24.6% or 5.8 million more than in 2020. These numbers demonstrate a clear link between renewed travel and loosened travel restrictions imposed due to the Covid-19 pandemic. Nonetheless, the Swiss figures remain low compared to pre-pandemic levels (Doyle, 2022), yet better than many of their European neighbors.

This ‘quick’ economic recovery in Swiss tourism can be explained by one substantial difference compared to other countries. Throughout the pandemic, as shown in Table 1, Swiss hotels were never obliged to close their properties completely; instead, they were obliged to close their restaurants and other points of sale or limit service capacity. Nonetheless, due to a dramatic drop in international travel through strict travel bans and ever-changing sanitary measures, some independent Swiss hotels decided it was economically unfeasible to remain open; thus, they temporarily opted to suspend their business activities. Further, the effects of the Covid-19 pandemic on the hospitality region were (and continue to be) substantial. The pandemic has potentially been the most significant crisis witnessed worldwide for all industries and stakeholders within those industries.

**Table 1.** Covid-19 waves in Switzerland.

Waves	Start date	End date
First wave	24 Feb. 2020	30 April 2020
(Intermediate wave)^a^	1 May 2020	Sept. 30, 2020
Second wave	1 Oct. 2020	14 Feb. 2021
Third wave	15 Feb. 2021	20 June 2021
Fourth wave^b^	21 June 2021	Oct 2021
Fifth wave^b^	Nov 2021	Present

Source: Adapted from Swiss Medical Weekly https://doi.org/10.4414/smw.2021.w30105

^a^The intermediate wave represents the relaxation of measures. There was little evidence to indicate that a second wave would arrive.

^b^These waves have been estimated to the month as no exact dates were found.

For the hospitality industry, the combination of a lack of tourists and the strict limitation of people in the same place at the same time (no more than 50 people on site) set by the Swiss government resulted in a constantly changing hotel environment. Over nearly 2 years, Swiss hoteliers have faced restrictions, relaxation of measures, and new restrictions again. Further, each adaptation needed to be communicated in ‘real-time’. While previous researchers on natural disasters focused on the responding and recovering stage, this pandemic obliged hotels to remain in the ‘responding’ stage by updating their websites and social media sites more frequently than any other period in history. The prolonged restriction and relaxation measures over the past 2 years have provided hoteliers time to learn, plan and execute crisis communication strategy, should they choose to do so. This leads to the overarching research question: How did the messages, specifically Situational Crisis Communication Theory (SCCT) strategies from Swiss hoteliers regarding Covid-19, evolve over the past 20 months?

This paper examines what messages and SCCT strategies Swiss 4 and 5-star hoteliers communicated to their stakeholders through their official websites from June 2020 to March 2022. The purpose is to analyze the evolution of the messages and the SCCT strategies that were used more effectively over time to better prepare them for what and how they could communicate in the next crisis. By investigating the messages through a prolonged period, we could detect organizational learning through the evolution of messages.

Our paper aims to make three theoretical contributions to studied in crisis communication: Firstly, while many previous studies have investigated communication during health crises in the tourism industry, they have predominantly focused on customers’ perceived image on social media, particularly Twitter (e.g. Carvache-Franco et al., 2022a; Carvache-Franco et al., 2022b) and Facebook (e.g. Salem et al., 2021). We have found scant literature that focused on the messages posted on hotels’ official websites, which represent the projected messages controlled by hoteliers. Secondly, most previous literature that applied SCCT focused on coding and analyzing SCCT strategy (Liu-Lastres et al., 2020) but did not incorporate thematic analysis. Incorporating thematic analysis and visualization enable the audience to view the most important themes and concepts, providing a different level of understanding. Finally, we have yet to find a comparable study that has tracked hotel messages over this length to determine the evolution of communication strategies to use in times of crisis, even though researchers have advocated a longitudinal approach (Salem et al., 2021). Many Covid studies addressed a specific period (in early 2020) (e.g. Carvache-Franco et al., 2022a; Carvache-Franco et al., 2022b; Salem et al., 2021). However, as shown in Table 1, the Covid-19 pandemic had a clear starting point but no ending place. Crisis offers a unique learning opportunity for organizations to improve their crisis management skills (Liu-Lastres et al., 2020). Thus, studying what was communicated during a crisis over a prolonged period may indicate the evolution of organizational learning as evidenced by the changes in messages.

Nonetheless, unlike previous studies that focused on one crisis in one company, our study applies SCCT on an industry level by analyzing the messages communicated by more than one property. This provides the opportunity to gauge if ineffective communication is systemic to an industry or if it differs from one property to another. On a practical level, we believe our study could contribute to more effective crisis communication by hoteliers when the next crisis hits, be it victim (like the pandemic), accidental, or preventable (Liu-Lastres et al., 2020). Thus, hoteliers could apply the same logic of this study when faced with another victim crisis but could also reflect on the appropriate strategies to apply for accidental and preventable crises. Research has shown that effective communication can reduce risk perception and long-lasting negative impacts on the property (Xie et al., 2021) while protecting its reputation (Kim et al., 2021). Thus, it is critical that hoteliers apply SCCT strategies in their messages during crises.

## Literature review

### Crisis management in tourism/hospitality industry

*Crises* are defined as unpredictable events that can impact an organization’s performance, damage organizational reputation, and generate negative outcomes (Coombs and Holladay, 2002; Cheng, 2018; Ki and Nekmat, 2014; Mukkamala et al., 2015). Crises are critical incidents or events that occur suddenly and without warning (Chen et al., 2021; Moerschell and Novak, 2020; Siddoo, 2021) with low probability yet high impact that threaten the viability of the organization (Canhota and Wei, 2021). The unpredictable and unexpected nature crisis can lead to feelings of concern, irrationality, and even shock (Coombs, 2007). While these feelings are natural during a crisis, they can increase the perception that a destination is not safe, thus making it less attractive to tourists.

In the tourism/hospitality industry, it is the responsibility of hoteliers to inform tourists and ensure their safety. Hotels must guarantee that the destination is perceived as safe by the tourists (Barbe and Pennington-Gray, 2018), as safety concerns are a significant predictor of travel intentions (Floyd et al., 2008) and affect their decision-making (Schroeder and Pennington-Gray, 2015) and travel behavior (Jingyi and Furuoka, 2020). According to Sano and Sano (2019), “effective crisis communication is required to restore consumers’ confidence in a destination when a potential or existing crisis has occurred” (p. 4).

For tourists, public perception is their reality; therefore, hotels need them to perceive that that destination is safe (Jingyi and Furuoka, 2020; Veil et al., 2011). Tourists are unlikely to visit places that they believe are unsafe (Sano and Sano, 2019). Whether a risk is existent or perceived, the perceptions of risk still have a bearing on the destination that is chosen as riskier destinations will be replaced by those perceived as less risky (Schroeder et al., 2013). Further, if tourist perceptions of risk increase, tourism demand can decline (Barbe and Pennington-Gray, 2018; Floyd et al., 2008; Schroeder et al., 2013). Nonetheless, tourists who had previously visited that destination reported they would not avoid that destination in the future as it enhances feelings of safety (Floyd et al., 2008). Tourists who visited the destination four times or more were more likely to return within 6 months of the event than less frequent visitors (Walters & Mair, 2012).

### Crisis communication

*Crisis communication* has been defined as “emergency messages intended to be instructional and informative, directed to the people at risk, the stakeholders, and the media” from pre-crisis to after the crisis has been resolved (Moerschell and Novak, 2020: p. 30). According to the literature, some communication during a crisis can be more positive than little or no information (Barbe and Pennington-Gray, 2018). When it is effectively done, the results are consequential. Consumers are reassured in their choices, and the risks associated with the crisis can be mitigated. According to Camilleri (2020), the better the communication, the more public approval a company may get and, subsequently, fewer negative consequences from the crisis. Yet, organizations must choose the crisis response strategy that is suitable for the type of crisis and the industry they belong to (Mukkamala et al., 2015).

For effective communication, the source credibility of the information is critical. Previous studies have posited that travelers seek information during crises from various sources. For example, many stakeholders seek crisis information from social media other than that provided by the company on their website (Berbecova et al., 2021; Sano and Sano, 2019). However, this information is hard to control (Sano and Sano, 2019) as many stakeholders hold different perceptions of the crisis itself. Further, social media can also complicate communication with an active exchange that may or may not be accurate (Berbecova et al., 2021). Misinformation can exacerbate the crisis by creating fear and panic (Moerschell and Novak, 2020), and inconsistent and disintegrated information after the crisis can harm a destination’s image (Berbecova et al., 2021). Virtual communities can provide mutual caring and social support during a crisis. They can be informative and therapeutic (Roth-Cohen and Lahav, 2021), but customers can create content and contribute negatively or positively to a brand (Azer et al., 2021). Thus, the message must be consistent across all channels employed by the organization. Suppose an organization communicates different messages and follows different paths sent by different people. In that case, the organization may lose all continuity leading to disorganization and chaos and hindering the ability to solve the problem (Moerschell and Novak, 2020) or address the crisis with verifiable information.

According to Sano and Sano (2019), during a high perceived risk like a pandemic, customers allocate higher source credibility to communications deriving directly from the organization rather than customer to customer (or social media) (Sano and Sano, 2019). Thus, websites have been perceived as more credible in high-risk crises like Covid-19 (where there is low organizational responsibility) than social media (which could be more effective in other types of crises with other attributions). Further, by publishing on their official websites, organizations can be timely and control this message (Zafra and Maydell, 2018). According to crisis communication literature, providing timely and accurate messages is crucial to reassuring customers (Berbecova et al., 2021) and mitigating the negative impacts of a crisis.

This leads to the question of how to communicate to reduce a crisis’s negative consequences. Traditionally, hotels were reluctant to communicate about events such as the Zika virus (or, potentially, Covid-19) if they believed that by mentioning the event, the risk perceptions of the tourists would increase (Barbe and Pennington-Gray, 2018). Tourists will not choose risky destinations and may avoid travel altogether (Xie et al., 2021). In previous health crises like the Zika virus, hotels used the crisis as an opportunity to inform guests on how to stay safe, i.e. buy mosquito repellent or find it in the hotel (Barbe and Pennington-Gray, 2018). For Covid-19, hygiene measures such as social distancing, masks, and hand sanitizer would be effective measures to communicate to guests and potential guests. However, the fact that many hotels post no messages or minimal communication online during a crisis could suggest something else; they may be concerned that talking about the crisis will heighten the tourists’ risk perceptions and, consequently, their intention to book rooms because of ‘perception of crisis responsibility is believed to be directly correlated to reputational damage’ (Coombs and Holladay, 2002: 173). Thus, hoteliers could be concerned that guests will be less likely to book their properties if they feel unsafe or at greater risk (Zizka et al., 2021). However, if hoteliers employ reassuring and effective crisis communication about the measures they are taking, they could foster consumer confidence and potentially improve the booking intentions of their guests (Kim et al., 2021).

### Situational crisis communication theory

As crises are considered negative events, stakeholders assess the crisis and attribute responsibility to an organization (Coombs, 2007). Coombs (1995) developed the situational crisis communication theory (SCCT) to help anticipate ‘how stakeholders will react to a crisis in terms of the reputational threat posed by the crisis’ (Coombs, 2007: 4). The SCCT features a situational approach and suggests that an effective crisis response should contain three components: (1) instructing information, (2) adjusting information, and (3) reputational management strategies (Coombs, 2017; Liu-Lastres et al., 2020).

*Instructing information* includes details of the crisis and factual information that is known at that time (Coombs, 2017; Ki and Nekmat, 2014). Nothing should be reported that has not been verified (Liu-Lastres et al., 2020). *Adjusting information* consists of listing the specific actions that are taken to react to the crisis (Coombs, 2017; Ki and Nekmat, 2014; Liu et al., 2018). During the pandemic, examples of adjusting information were given as sanitary measures or directives customers needed to follow to stay safe. However, adjusting information also includes one significant criterion of effective crisis communication, i.e. empathy (Jong, 2020; Wong et al., 2021; Xie et al., 2021). During the various stages of the pandemic, customers expected to see a ‘care’ response from hoteliers that focused on empathy instead of an emphasis on selling rooms or getting people back at all costs (Azer et al., 2021). The actual response to the crisis combines instructing and adjusting information. It can lead to the implementation of *reputational management strategies* such as denial (i.e. admitting no blame), diminishment (i.e. making excuses), rebuilding (i.e. apologizing), and bolstering (i.e., counter-balancing negative perceptions of the crisis) (Barbe and Pennington-Gray, 2018; Coombs, 2017; Ki and Nekmat, 2014; Liu et al., 2018).

However, before considering SCCT strategies for reputation management, the first priority is protecting stakeholders from harm, not protecting the organization’s reputation (Coombs, 2007; Kim and Liu, 2012; Wong et al., 2021). Thus, when a crisis affects public safety, such as the Covid-19 pandemic, instructional information on protecting ourselves is more important and should come before addressing reputational concerns (Coombs and Holladay, 2002; Coombs, 2007; Kim and Liu, 2012; Liu-Lastres et al., 2020; Stewart and Young, 2018).

According to SCCT, organizations fall into one of three clusters in times of crisis (Liu-Lastres et al., 2020). When the organization has little to no attribution for crisis responsibility from the stakeholders, it is called a victim cluster. In a victim crisis, harm is inflicted on the organization and stakeholders (Liu et al., 2018), and the organization has very weak responsibility (Coombs, 2007). The second cluster is called an accidental cluster. In accidental crises, there is minimal attribution as the organization has unintentional or uncontrollable actions such as technical error, accident, or product harm (Coombs, 2007).

The third cluster, i.e. preventable or intentional, is potentially the most damaging to an organization and its reputation. Preventable crises occur when the organization intentionally places stakeholders at risk by knowingly violating laws or regulations, not preventing an accident or defective products from reaching markets the organization has strong attributions of crisis responsibility such as a human-error accident, product harm, or organizational misdeed (Coombs, 2007). These clusters and the attribution of responsibility affect the amount of information needed to reassure customers that the company is still trustworthy.

Previous crisis communication literature defines the crisis into three distinct phases: Pre-Crisis, Crisis Response, and Post-Crisis. The Pre-Crisis phase includes day-to-day operations and the positive relationships with stakeholders that should be built or enhanced during this time. Pre-Crisis is an ideal moment to brainstorm potential crises, prepare or adapt crisis plans, and train for the most probable crises. It is also a time to create a dark website (Chen et al., 2021; Moerschell and Novak, 2020; Siddoo, 2021). Unfortunately, the Pre-Crisis phase is not always done effectively due to a lack of time, resources, or forward thinking.

Once the crisis hits, organizations must focus on the actions and responses to mitigate the effects of the crisis. The Covid-19 pandemic appeared as an unprecedented global crisis that was exacerbated by international travel (Canhota and Wei, 2021). It has proven more complex and resilient than any previous crisis (Canhota and Wei, 2021). During the Crisis phase, organizations consider what they need to communicate and how they will do so. Beyond the base response, which is the automatic response for all crises, a pandemic of this reach necessitated further SCCT strategies.

The Post-Crisis phase occurs after the immediate threat is resolved and the danger has passed. This is the time for audits and assessments on how well the organization responded to the crisis, how much reputational damage was mitigated, and how much reputational capital was lost (Chen et al., 2021; Moerschell and Novak, 2020; Siddoo, 2021).

During the ‘recovery’ from a sanitary crisis like the pandemic, organizations can attempt to reverse the negative impact on the local area (Chen et al., 2021). Table 2 summarizes the original SCCT strategies and the evolution of the strategies over time. The messages from the Swiss hotels will be linked to the strategies chosen for a victim crisis with little to no responsibility.

**Table 2.** SCCT strategies.

Attack the attacker (or accuser): Confronts group or person that claims crisis exists	(Coombs, 1995)
Denial: denies the crisis exists	(Coombs, 1995)
Excuse: attempts to minimize organizational responsibility for crisis	(Coombs, 1995)
Victimization: reminds stakeholders that organization is a victim of crisis	(Coombs, 1995)
Justification: minimizes the perceived damage inflicted by the crisis	(Coombs, 1995)
Ingratiation: praises stakeholders and reminds them of the past good works done by the organization	(Coombs, 1995)
Corrective action: tries to prevent a repeat of the crisis and/or repair damages done	(Coombs, 1995)
Full apology: publicly accepts responsibility for the crisis/asks forgiveness	(Coombs, 1995)
Scapegoating: shifts the blame to another person/group outside of the organization	(Ki and Nekmat, 2014)
Mortification: needs to apologize for an act	(Cheng, 2018)
Enhancing: focuses on their current good deeds	(Cheng, 2018; Kim and Liu, 2012)
Transferring: uses a credible third party’s crisis response to transfer that third party’s credibility onto themselves; can be done by citing credible 3^rd^ parties like world health organization (WHO) or Centers for Disease control and Prevention (CDC) regarding hygiene practices	(Cheng, 2018; Kim and Liu, 2012;Schroeder and Pennington-Gray, 2015)
Ignoring: where organizations implicitly state that a crisis does not exist by disregarding the crisis	(Kim and Liu, 2012)
Renewal: replacing defensive discourse with optimistic rhetoric to the post-crisis phase (e.g. stakeholder commitment, commitment to correction, and core values)	(Ulmer and Sellnow, 2002)

By tracking the messages from the same Swiss hotels over 2 years, we attempt to address how hoteliers communicated about a crisis over time and what SCCT they employed in three distinct periods of the pandemic. Our objective is to provide practical application of these results to be reflected upon when this or another type of crisis affects the industry in the future.

## Methodology

This study chose a Swiss hotel sample of 4 and 5-star independent hotels for several reasons. Firstly, unlike chain hotels where the messages derive from company headquarters, we were interested in independent hotels that would be obliged to communicate their own original messages. Secondly, we chose 4 and 5- star hotels as they have, in general, more financial resources to invest in corporate communication. Finally, Swiss hotels were selected as they did not close at any point during the pandemic, thus making a longitudinal study possible.

While Swiss hotels are located in various regions and are often set to the local language of the canton in which the hotel is situated (French, German, or Italian), most of these websites had a combination of the national languages, and all of them had an English language setting. For consistency, the messages communicated in English were gathered from the official hotel websites, and the search for Covid-19 information was conducted. The homepage of each hotel website was scrutinized for direct messages that could be found. Further, each website was searched using the keywords’ Coronavirus’ and ‘Covid-19’, which were entered into the search boxes when available. The website content from individual hotel websites was copied and merged to create a master Website file.

We have chosen three critical moments of the pandemic: The beginning of the crisis, i.e. the first few months that led to a series of unprecedented sanitary measures; the middle, i.e. a year later with vaccination available to the general public and the constant adaptation to sanitary restrictions; and the current state, i.e. the most recent messages (until March 2022) when all establishments have returned to business as ‘normal’. In this study, we refer to these three time frames as Snapshot 1 (June 2020), Snapshot 2 (June 2021), and Snapshot 3 (February 2022).

The messages on the official hotel websites from June 2020 to March 2022 were mined by two research assistants. The process of analysis involved two stages. Firstly, the raw data collected from hotels’ websites was cleaned to allow for uniform processing. The cleaning process involved homogenizing formats (e.g. different capitalizations or inconsistent paragraphing). Names and job titles were also removed under the condition that they were not embedded in relevant sections of the text. The output was formatted in sentence case, with one paragraph corresponding to one website section or message.

Secondly, co-occurence and thematic analyses (Chia and Xiong, 2022; Kajla et al., 2022; Salari and Murphy, 2022) were conducted using Leximancer software (edition 5.0). The default setting was used for concept seed identification, then similar concepts (e.g. area and areas, guest and guests, restaurant and restaurants, room and rooms, mask and masks) were merged. The visualization was set to present 90% of concepts/themes.

### Leximancer

Leximancer uses statistics-based algorithms to analyze text automatically and visually displays the selected information in the form of concept maps (Smith and Humphreys, 2006). Leximancer conducts semantic and relational extraction (Chan and Saikim, 2021). First, Leximancer calculates word occurrence and co-occurrence frequency to establish Concepts and expands Concepts to a thesaurus (Chan and Saikim, 2021). Second, Leximancer re-classify the documents based on Concepts and thesaurus and forms Themes (Chan and Saikim, 2021). The Concepts (or Themes) are contextually clustered according to weight and relationship to create a Concept (or Theme) cluster map. The map is based on frequency and similarity and illustrates the Concepts (or Themes) sharing a topic theme in the same color as their cluster group circle and cluster label (Leximancer, 2021; Sotiriadou et al., 2014). The essential Concept (or Theme) is assigned the color red. Then in descending order of significance, the remaining Concepts (or Themes) are identified by orange, yellow, green, blue, and purple (Tseng et al., 2015).

Once the data was mined from the official websites and divided into the three snapshots of the study, the comments were manually coded to link each comment to a specific SCCT strategy or strategies. The longer the message, the more strategies were potentially identified. This coding was done individually by each of the researchers before meeting to compare the results. Any discrepancies were discussed until an agreement was met. The results for the Leximancer and the SCCT strategies can be found in the next section.

## Results

### Leximancer at three key points

**Figure 1.**
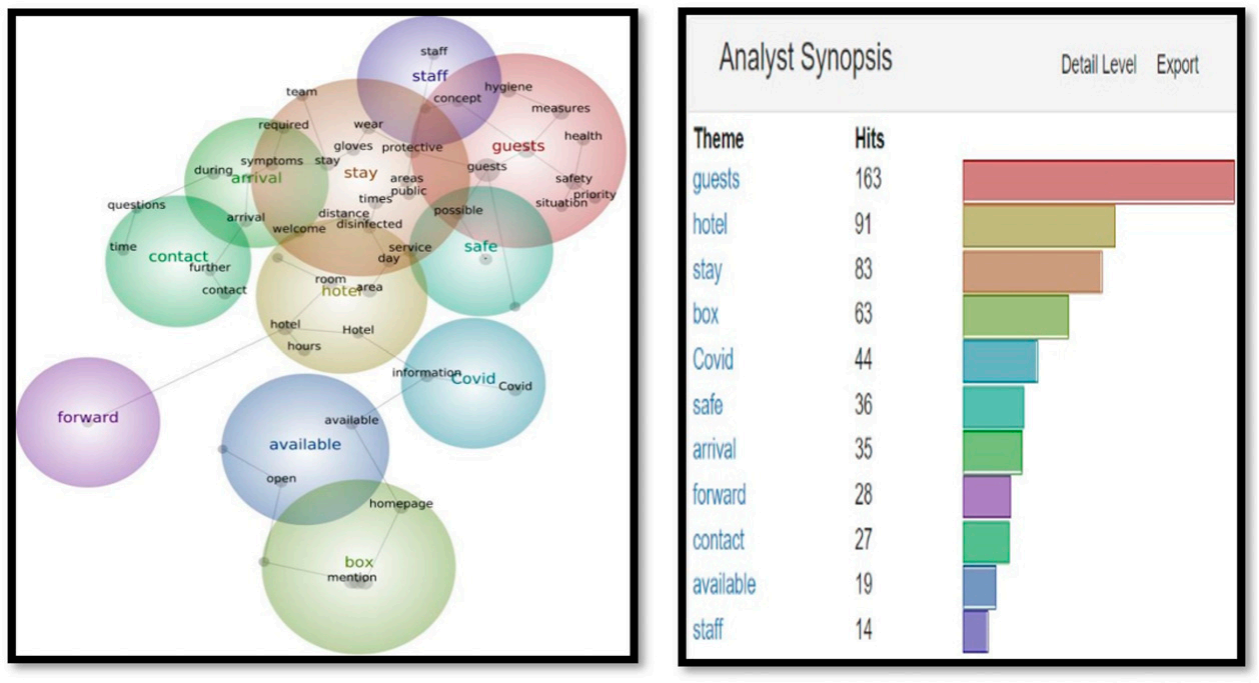
Snapshot 1: June 2020. Themes.

**Figure 2.**
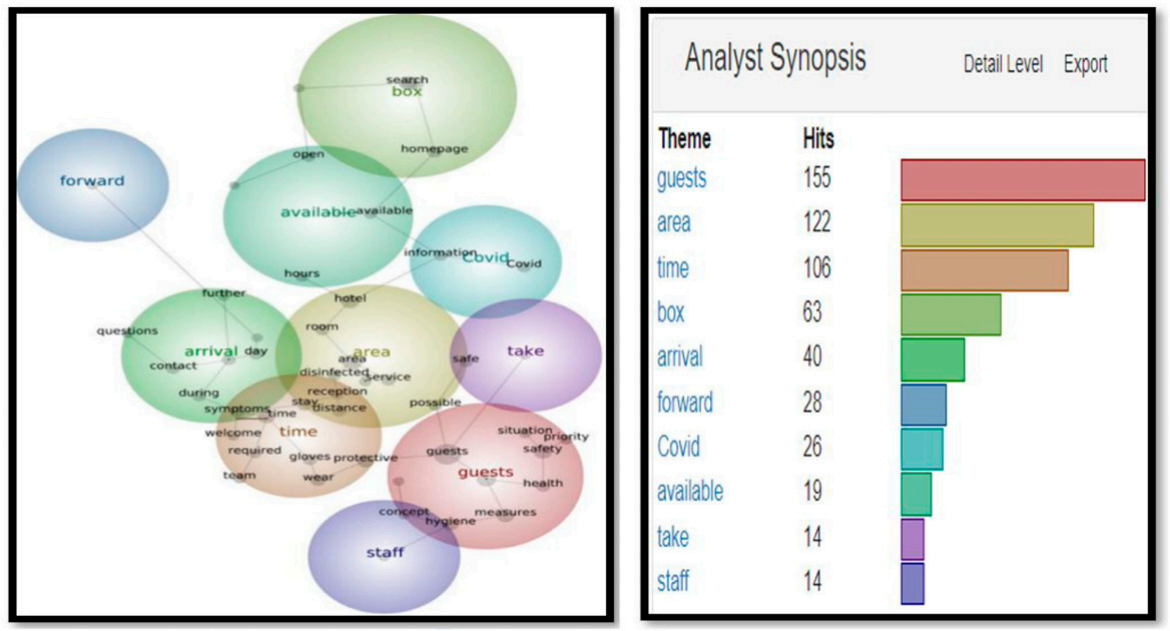
Snapshot 1: June 2020. Concepts.

**Figure 3.**
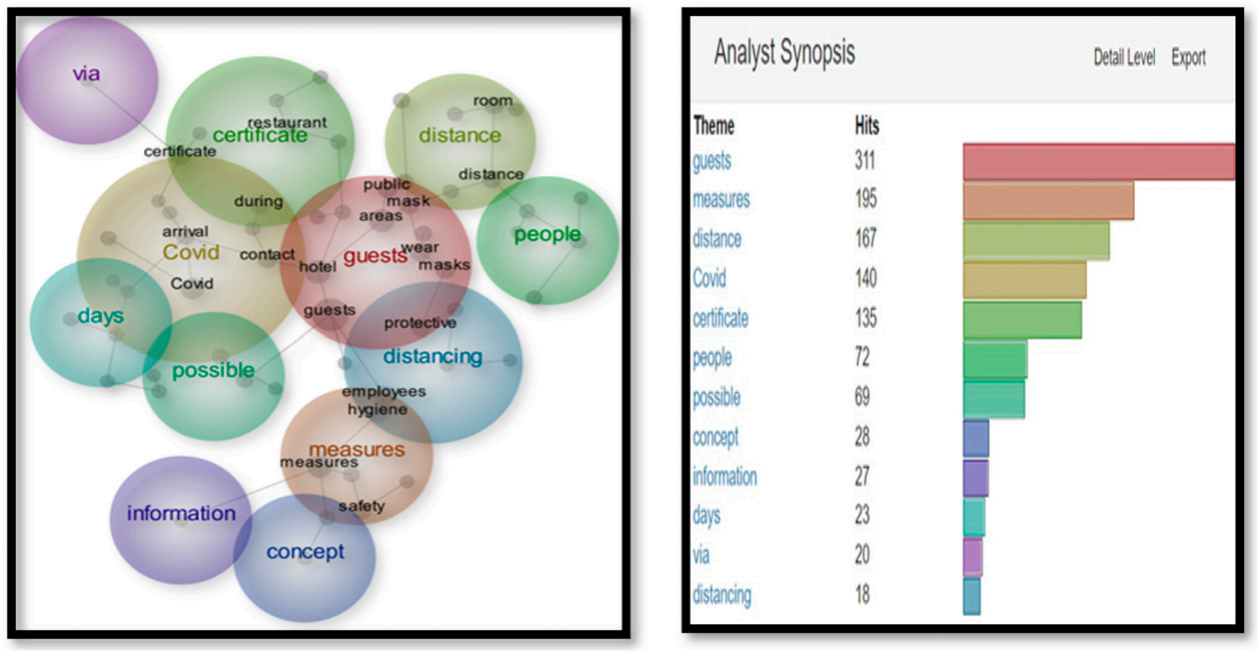
Snapshot 2: 1 year later June 2021. Themes.

**Figure 4.**
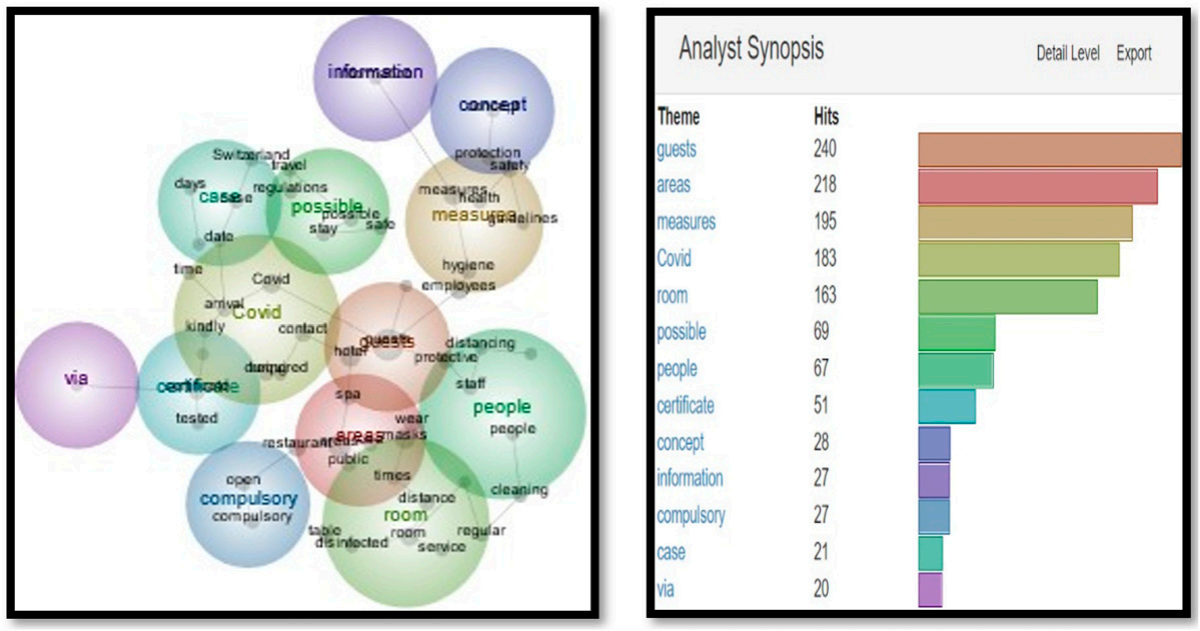
Snapshot 2: 1 year later June 2021. Concepts.

As seen in Figure 1, themes such as ‘guests’ (163 hits), ‘hotel’ (91 hits), ‘stay’ (83 hits), and ‘box’ (63) top the list. These are in warm colors as per the Leximancer analysis to demonstrate the frequency of their use. According to Figure 2, the most common concepts are ‘guests’ (155 hits), ‘area’ (122 hits) and ‘time’ (106 hits). The specific term Covid appeared 44 and 26 times respectively Figure 3 and Figure 4.

**Figure 5.**
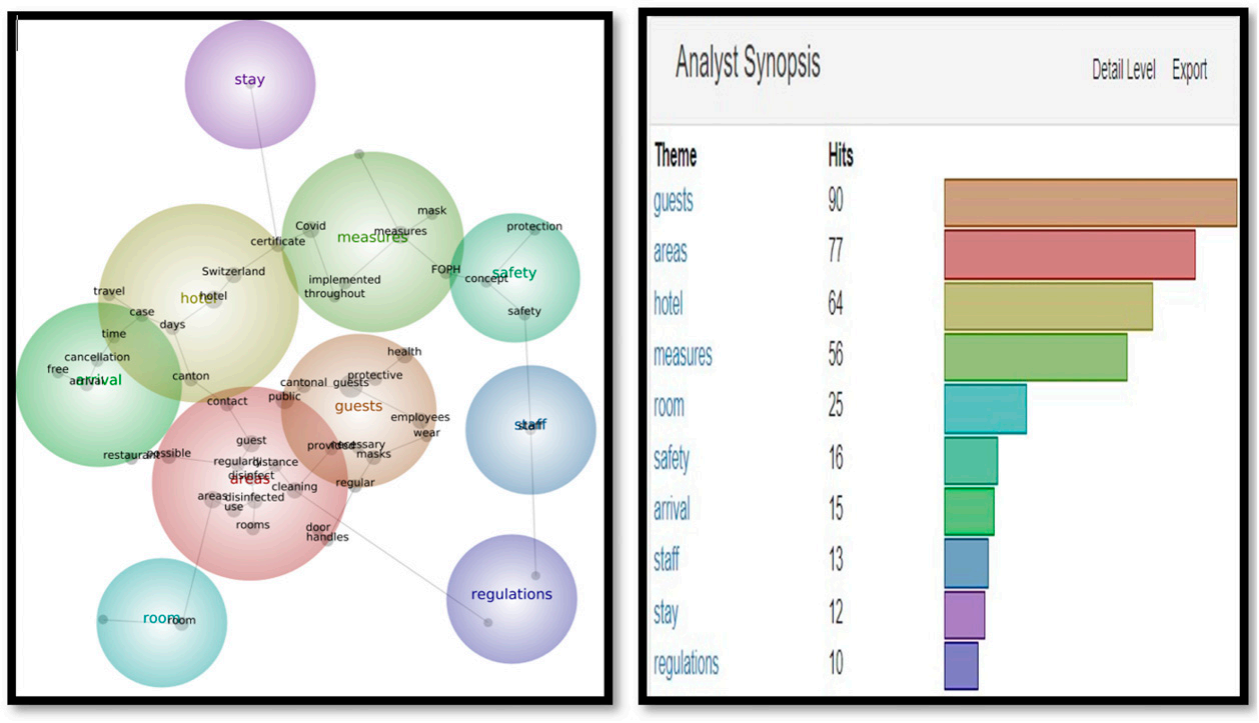
Snapshot 3: Back to normal. February 2022. Themes.

**Figure 6.**
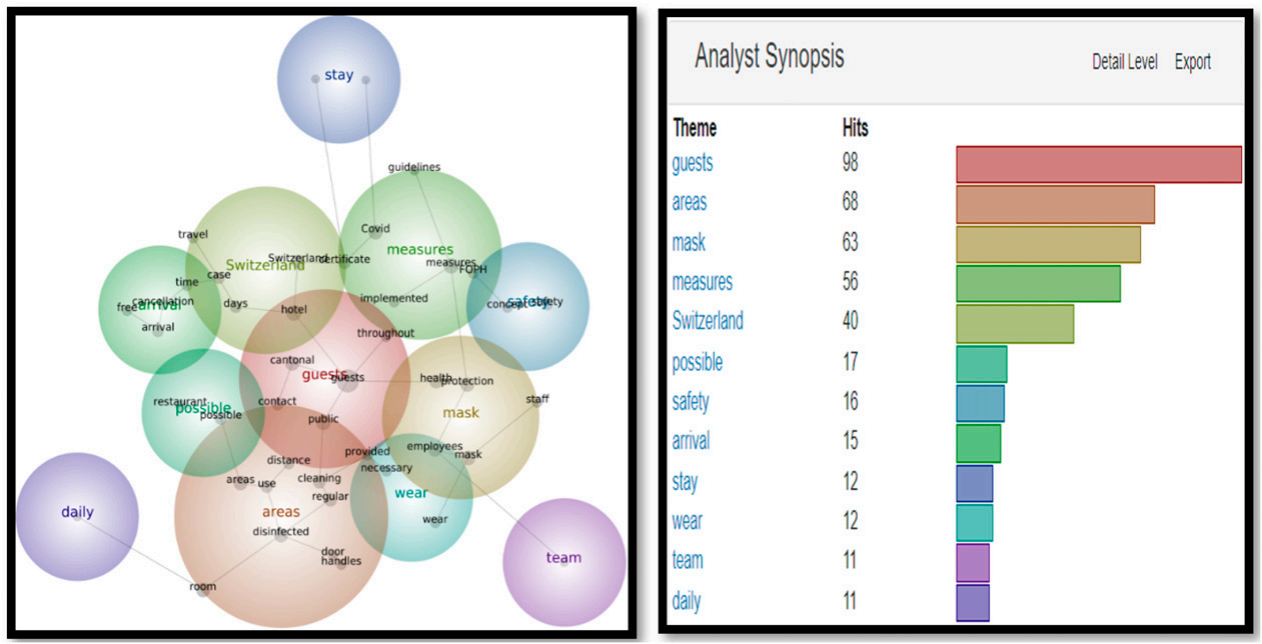
Snapshot 3: Back to normal. February 2022. Concepts.

In Snapshot 2, the key themes and concepts shifted**.** While ‘guests’ were still the most often cited as themes and concepts, (311 and 240), ‘measures’ and ‘Covid’ also appeared in the top four. Unlike Snapshot 1, where Covid was in a cool blue, in Snapshot 2, Covid was displayed in orange and yellow. A term like ‘certificate’ did not appear at all in Snapshot 1, while it displayed 135 (green) and 51 times (blue) in Themes and Concepts. This would be the only snapshot in which the certificate was frequently mentioned to make into the Themes and Concepts (as seen in Figures 5 and Figure 6).

The visual for Snapshot 3 (as seen in Figures 5 and 6) included ‘guests’, ‘areas’, and ‘measures’ in the top four hits for Themes and Concepts. Interestingly, ‘mask’ appeared first time in the Concepts, while ‘certificate’ and ‘Covid’ disappeared from the list.

### SCCT strategies

Of the potential 14 SCCT strategies, seven (*ingratiation, corrective action, transferring, victimization, enhancing, justification,* and *renewal*) were used in each period of this study. Strategies more appropriate for accidental crises (*full apology, mortification*, or *denial*) or preventable crises (*scapegoating, excuse, attack the attacker*, or *ignoring*) were not used by Swiss hoteliers when communicating about the Covid-19 pandemic, a victim crisis. Table 3 summarizes the SCCT strategies during the past 2 years.

**Table 3.** SCCT strategies during the Covid-19 pandemic.

Strategy	Snapshot 1, %	Snapshot 2, %	Snapshot 3, %	Total
Ingratiation	29	25	33	87%
Corrective action	23	21	21	65%
Transferring	19	21	24	64%
Victimization	14	3	2	19%
Enhancing	10	4	2	16%
Justification	3	17	15	35%
Renewal	3	9	3	15%
Total	100	100	100	

According to Coombs (2017), it is not necessary to offer more than a base response, i.e. instructing and adjusting information, including corrective actions, in a victim crisis. Nonetheless, as seen in Table 3, Swiss hoteliers also included other SCCT strategies. Of all of the supplemental strategies, ingratiation was used most often. In other words, hoteliers praised stakeholders and reminded them of the good work done in the past by the hotels (Liu-Lastres et al., 2020). This could imply that hoteliers wanted to create a bond with their guests and leverage their built reputation.

While corrective actions were more numerous in the texts, each message that included a list of corrective actions counted as one SCCT strategy. For example, in Snapshot 1, the number of corrective actions cited in Swiss hoteliers’ website messages ranged from one corrective action to 71 corrective actions. For Snapshot 2, the numbers varied from one to 116 corrective actions; for period 3, the corrective actions ranged from one to 93. The number of corrective actions coincided with the ever-changing measures imposed by the Swiss government and/or the local area where the hotels were situated. In Switzerland, the independent cantons can decide what measures to act upon and what to refuse. For example, the Swiss Italian part was hit heavily by the Covid-19 pandemic (as it is the closest geographically to Italy, which was one of the hardest-hit areas in Europe). By Snapshot 3 (as seen in Figures 5 and 6), when other cantons were relaxing their measures and reducing the corrective actions they took, Swiss Italian hotels had the highest number of actions still in place (93).

*Transferring* is the only strategy with growing importance (from 19 to 21 to 24%). The increasing importance of transferring may indicate that hoteliers had counted on the government or health authorities to convince guests that implementing some sanitation and social distancing rules is not their choice but demanded by the authorities. The Covid-19 pandemic is a victim crisis, but hoteliers only used victimization in Snapshot 1 and dramatically moved away from this strategy in Snapshots 2 and 3. This change also demonstrates the importance of observing hoteliers over a prolonged period to detect changes in their strategies.

## Discussion

In response to the overarching research question: How did the messages, specifically SCCT strategies from Swiss Hoteliers regarding Covid-19, evolve over the past 20 months?

Table 3 shows that the five key strategies used are *ingratiation, corrective action*, *transferring, victimization*, and *justification*, but their use varied during the past 20 months. *Ingratiation* was the most popular strategy for the three studied periods (29, 25, and 33%, respectively). *Corrective action* (23, 21, and 21%, respectively) and *transferring* (19, 21, and 24%, respectively) were the second and third most popular strategies and may suggest that hoteliers leverage authorities explain the corrective actions.

*Justification* was not important in Snapshot 1 (3%) but gained importance in Snapshot 2 and 3 (17%, and 15%, respectively). This could be explained by the lack of knowledge of what hoteliers needed to do to keep their customers safe initially during a pandemic. Justification may work with corrective action and transferring to justify the implemented corrective actions. While Swiss hotels positioned themselves as victims in the early period of the pandemic, it was not a sustainable strategy to keep. As all industries were affected by the Covid-19 pandemic, trying to place themselves as ‘victims’ to potential tourists who were all victims in their own rights was not an effective long-term strategy.

Similar to *victimization* (14, 3, and 2%, respectively), *enhancing* was used more in Snapshot 1 than in Snapshots 2 and 3 (10%, 4%, and 2%, respectively). Swiss hoteliers could not rely on being a victim or past actions to bolster their reputation during a crisis; instead, they needed to promote their current good deeds, some of which did not exist before the pandemic. On the other hand, *renewal* was used more in Snapshot 2 than in Snapshot 1 and 3 (3, 9, and 3%, respectively). Renewal focuses on positive rhetoric, and hoteliers were able to adapt their messages accordingly.

As the pandemic continued, Swiss hoteliers became savvier about how to deal with the changing measures; thus, their communication strategies could extend beyond base information to confidence in how they were dealing with the situation. In Table 4, some of the specific comments deriving from the Swiss hotels have been provided as linked to the specific SCCT strategies.

**Table 4.** SCCT strategies- hotel website comments.

Strategy	Snapshot 1	Snapshot 2	Snapshot 3
Ingratiation	We thanks you for your support, your understanding, and your loyalty…	Dear valued guest, friend, and partner, we continue to prioritize your well-being	We would like nothing more than for you to feel safe and at home with us during these extraordinary times…
Corrective action	We would like to inform you of the current status of the new offer…	Coronavirus health and safety measures are available here…	Here you with find the list of all of the safety measures implemented
Transferring	On Monday, 16th March 2020, the Federal Council declared the ‘extraordinary situation’…	We are strictly following the rules and regulations of the Federal Council and the WHO…	This ensures that the guidelines of Hotelleriesuisse, the Federal Office of Public Health, and WHO are implemented…
Victimization	The current situation requires certain restrictions and measures beyond our control…	Unfortunately, this is the only way to keep everyone safe from the Coronavirus…	There are many things we cannot and are not allowed to do…
Justification	For safety reasons, appointments can only be made by prior agreement…	For your personal health and safety and that of our employees, the following measures have been taken…	So that everyone feels comfortable and safe, we are following the strong Swiss regulations…

Generally, Swiss hoteliers focused on what they were doing beyond simply corrective actions. They communicated their appreciation to the stakeholders for their patience and fidelity during a crisis (i.e. ingratiation). Further, they focused on enhancing by communicating what they are doing to help their employees (during and after confinement) or what they are doing to help the community. As confirmed in this study and previous literature (Cheng, 2018; Kim and Liu, 2012; Schroeder and Pennington-Gray, 2015), Swiss hoteliers often cited credible Swiss government officials or health sites to transfer their credibility onto themselves (i.e. transferring). While Covid-19 falls under the victim cluster, the SCCT strategy of ‘victimage,’ i.e. reinforcing the belief that organizations deserve sympathy (Coombs, 2007), was ineffective.

During the pandemic, Swiss hoteliers communicated their cleaning standards, rules and regulations, booking options and cancellation policies, and other safety procedures (Siddoo, 2021), but they also began to link their service quality to the importance of cleanliness and safety (Jiminez-Barreto et al., 2021; Kim et al., 2021). Many hotels installed new cleaning technology (Salem et al., 2021). As the pandemic posed a health risk, stricter cleaning policies and perceived professionalism (Jiminez-Barreto et al., 2021) were emphasized and communicated. Most Swiss hotels in this study posted about hygiene, safety, health, and protective measures (providing information to guests, i.e. the base response of SCCT) (Zizka et al., 2021). However, beyond the protective measures, as the 2 years went on, Swiss hoteliers shifted to more upbeat messages to get people back (Zizka et al., 2021). It seems their strategies paid off when studying the most recent tourism numbers.

In addition to analyzing SCCT strategies adopted by Swiss hoteliers, this study also analyzed Themes and Concepts in three snapshots. Overall, the most important themes are ‘guests’ and ‘areas’. However, the evolutions of Themes and Concepts provide further insights. For example, ‘Measures’ did not appear in Snapshot 1 when there was unpredictability facing an unprecedented pandemic; but appeared in Snapshot 2 and 3, when governments and health organizations had already developed guidelines and vaccines. The availability of vaccines initiated the debate on vaccination, and the necessity of a certificate, as shown in Snapshot 2. However, the certificate disappeared when the mask appeared in Snapshot 3 (as seen in Figures 5 and 6). These findings demonstrated the importance of monitoring and tracing the evolution of crisis communication over time.

Our study demonstrates a clear evolution of crisis communication over 20 months. The length of the pandemic allowed hoteliers the time to reflect and learn about the situation and draft more effective crisis messages. When looking back at this extraordinary period of hospitality, future generations will recognize this as the “annus horribilis” for tourism. Nonetheless, the lessons learned from the crisis are consequential. The crisis will happen again. Innovative organizations develop their organizational resilience through lessons learned from each crisis. However, these lessons can only be learned through reflection in the post-crisis phase. While hoteliers are racing to return to ‘normal,’ will they take the time to do the thorough post-crisis reflection that is primordial when moving forward?

## Conclusions

The literature shows that the tourism industry is highly susceptible to crises. However, it has high resilience and a relatively quick recovery from the impacts of these events (Berbecova et al., 2021), particularly when the responses are proactive (Yeh, 2021). In studying the same group of hotels over 20 months of the Covid-19 pandemic, we traced an evolution of communication practices and SCCT strategies over time. This study identified ingratiation, corrective action, transferring, victimization, and justification as the five key strategies used during the crisis. This finding, together with thematic analysis and visualization, demonstrated the evolution of crisis communication. We identified the changes in themes, concepts, and vocabulary richness through the three snapshots. Hoteliers must ensure that all guests, employees, and local stakeholders are safe and secure when doing business with them. Management in all industries should be making strategic decisions to plan for future crises while limiting communication gaffes. These results and reflections can be used to prepare for the next wave or next crisis.

## Implications

### Theoretical implications

This study contributes to crisis communication and SCCT literature in three specific ways. Firstly, no literature to our knowledge addresses the evolution of website messages in Switzerland or elsewhere during an extended crisis period. Unlike studies that analyze and interpret messages from company websites at a fixed moment, we have seen the value of looking at the same properties with the same parameters on several occasions. The originality of analyzing messages on the industry, not individual or destination level, could be advantageous when making strategic decisions on a macro-scale. Secondly, this study investigated the SCCT strategies with thematic and co-occurrence analyses, which provide different levels of understanding of crisis communication. The coding and analysis of SCCT strategies at the sentence level provide a granular grasp of the messages and SCCT strategy applications. Alternatively, the thematic analysis and visualizations provide a bird’s-eye view of the main topics at three specific periods during the crisis. The research method of combining message coding, thematic analysis, and visualization can be encouraged and adopted for future research. Further, these messages were analyzed during a ‘victim’ crisis. As seen in the literature, a victim crisis does not necessarily need more than a base response; however, as seen in this study, the gravity and long-term reach of the pandemic obliged these hoteliers to utilize more SCCT strategies than usual and to adapt them as time passed. This study identified the SCCT strategies used and the relative usages of these strategies, as shown in Table 3. Hence, the research presented the evolution of SCCT strategies over a prolonged period.

### Managerial implications

There are several practical implications derived from our study. Firstly, we encourage practitioners to examine their posted messages in the past 2 years, analyze the SCCT strategies used, and benchmark their results against our research findings in Table 3. This reflection is part of organizational learning which can build up resilience for the future.

Secondly, when faced with a crisis, the base response is primordial, with a particular emphasis on empathy. Like previous studies in many industries, Swiss hoteliers did not consistently communicate empathy. Hotel managers must focus on including empathy in their crisis messages.

As seen in our study, in a victim crisis such as the Covid-19 pandemic, the five SCCT strategies that were the most effective were ingratiation, corrective action, transferring, victimization, and justification. Nonetheless, hoteliers should consider utilizing enhancing and renewal as well, as both of these strategies provide positive messages that can be associated with the company. Above all, managers must match the SCCT strategies to the type of crisis they are facing; there is no one ‘correct’ way to respond; instead, the response should be based on a reflection of crisis type, attribution, and reputation management.

While our study focused on the hospitality industry, given the scant research on crisis communication for a prolonged period for the hotel industry, this study could serve as a reference for other tourism-related industries. Finally, this study may inspire hoteliers when faced with other types of crises, i.e. accidental or preventable.

### Limitations and future studies

This study examined the messages over 2 years from Swiss 4 and 5-star independent hotels, yet some limitations existed. First, the sample size of 48 is small compared to the 222 independent hotels that were initially targeted. Only these 48 continued to publish Covid-19-related messages on their websites. Many hotels remained silent. On the other hand, the fact that we could trace these same hotels at three specific times over 2 years has helped us establish a pattern in crisis responses over an extended period. This is one of the contributions of our study. A future study could examine the messages of other geographical areas or other types of hotels.

A second limitation is the use of website messages alone. While the original intention was to track the messages on the official websites and social media pages, the disparate use of social media over the entire study period led to potential biases in the results. For this reason, social media posts were excluded from this study. A future study could investigate social media messages posted by hotels in times of crisis.

However, social media may have been problematic due to the third limitation. We only examined messages in English, although Switzerland has four official languages: French, German, Italian, and Romansch in the three geographical areas of Switzerland (Swiss Romand, Swiss Italian, and Swiss German). It may seem illogical that we did not use their official languages; however, Switzerland is reliant on tourism that derives from other areas. Not all Swiss speak all languages, but all Swiss study English in school. Further, for international travelers, i.e., most tourists, the common language is English. Finally, while the Swiss hotel websites offer a version in the language of the region where they are located, they do not often offer all national languages; instead, they offer the language of their region and English. Thus, for consistency, we gathered all of the messages in English. A future study could investigate the differences between the regions in Switzerland.

### Current state of hospitality industry

While this study captured the messages in three snapshots over 2 years, it was a unique moment for Swiss tourism that will not be replicated. We studied what and how Swiss hoteliers communicated regarding an unprecedented moment in time. Unlike other crises and equally problematic for all industries, there was no previous SCCT example to base their response on to gauge if they communicated effectively. While conducting this study, there have been five waves of the pandemic, and subsequently, the hospitality industry has been in constant evolution. Researchers have begun analyzing the effects of the Covid-19 pandemic on all industries, including the hospitality industry (Azer et al., 2021; Jong, 2020; Wong et al., 2021; Xie et al., 2021; Zizka et al., 2021). The initial ideas linked to effective crisis communication, that of reassuring customers that they are safe and secure, remain consistent recommendations, but the importance of empathy has been reinforced (Azer et al., 2021; Jong, 2020; Wong et al., 2021; Xie et al., 2021; Zizka et al., 2021). Researchers agree that this crisis can be an opportunity to communicate with stakeholders and display leadership to reduce the adverse effects of the Covid-19 pandemic (Wong et al., 2021; Zizka et al., 2021). Moving forward, hoteliers will not be able to predict the next crisis or when it will be. However, through the Covid-19 pandemic, they have certainly had the opportunity to hone their crisis communications strategies over the past 2 years. The question remains: What message and SCCT strategy will be used when faced with the next crisis?
